# Clinical significance of elongation factor-1 delta mRNA expression in oesophageal carcinoma

**DOI:** 10.1038/sj.bjc.6601941

**Published:** 2004-06-15

**Authors:** K Ogawa, T Utsunomiya, K Mimori, Y Tanaka, F Tanaka, H Inoue, S Murayama, M Mori

**Affiliations:** 1 Department of Molecular and Surgical Oncology, Medical Institute of Bioregulation, Kyushu University, Tsurumihara 4546, Beppu 874-0838, Japan; 2 Department of Abdominal Surgery, Saitama Cancer Center, Saitama, Japan; 3 Department of Radiology, University of the Ryukyus, Okinawa, Japan

**Keywords:** oesophageal neoplasms, quantitative real-time PCR, prognostic factors, elongation factor-1 delta, proto-oncogene

## Abstract

Elongation factor-1 (EF-1) delta is a subunit of EF-1, which is a protein complex that participates in the elongation step of mRNA translation and has recently been considered to correlate with oncogenic transformation. However, there has been no information regarding the clinical significance of EF-1 delta mRNA expression in malignant tumours, including oesophageal carcinoma. Thus, we quantitated the expression of EF-1 delta in malignant and benign oesophageal tissues and associated these levels with clinicopathologic parameters of oesophageal carcinoma. Paired oesophageal tissue samples from cancerous and corresponding noncancerous parts were obtained from 52 patients who underwent curative oesophagectomy. Quantitative analyses of EF-1 delta expression were performed using real-time quantitative reverse transcription–polymerase chain reaction. Elongation factor-1 delta mRNA overexpression in cancerous tissues compared to normal counterparts was observed in 38 of 52 (73%) patients. The mean expression level of EF-1 delta mRNA in cancerous tissues was significantly higher than that in noncancerous tissues (*P*<0.01). A higher expression of EF-1 delta was significantly correlated with lymph node metastases (*P*<0.05) and advanced stages (*P*<0.05). Furthermore, the cause-specific survival of patients with a higher expression of EF-1 delta was significantly poorer than those with a lower expression (5-year cause-specific survival rates; 23 and 54%, respectively, *P*<0.05). The results of this study indicated that EF-1 delta mRNA expression was significantly higher in cancerous compared to noncancerous oesophageal tissues, and a higher expression of EF-1 delta mRNA was correlated with lymph node metastases, advanced disease stages and poorer prognosis for patients with oesophageal carcinoma.

Elongation factor-1 (EF-1) delta is a part of the EF-1 protein complex that mediates the elongation step of protein synthesis by transferring aminoacyl-t RNA to 80S ribosomes fuelled by the hydrolysis of guanosine 5′-triphosphate (GTP) (Riis *et al*, 1990; Proud, 1994). Elongation factor-1 is composed of four subunits: alpha, beta, gamma and delta. Elongation factor-1 comprises the following distinct functional domains: a nucleotide-binding domain (EF-1 alpha) and a nucleotide-exchange protein complex (EF-1 beta/gamma). Elongation factor-1 delta also possesses a nucleotide-exchange activity and interacts with EF-1 alpha and EF-1 beta/gamma. The N-terminal domain of EF-1 delta contains the six-residue leucine-zipper motif typically seen in transcription factors, the function of which is not known (Sanders *et al*, 1993).

Considerable evidence suggests that translation factor activity plays an important role in regulating cellular growth, and several studies have provided a link between translation factor activity, cellular senescence and the expression of a number of oncogenes (Sonenberg 1993; Anand *et al*, 2002; Zimmer *et al*, 2002). Abnormal expression of translation factors such as EF-1 was shown to have dramatic effects on cellular growth, including transformation and tumorigenesis. We previously reported the clinical significance of EF-1 gamma, and overexpression of EF-1 gamma mRNA was correlated with the aggressiveness of gastric and oesophageal carcinomas (Mimori *et al*, 1995, 1996). Furthermore, elevated levels of EF-1 alpha and EF-1 gamma were also found in cancers of the pancreas, colon, breast, lung, prostate and stomach relative to normal tissues (Chi *et al*, 1992; Grant *et al*, 1992; Lew *et al*, 1992; Ender *et al*, 1993; Mathur *et al*, 1998; Su *et al*, 1998).

However, to the authors' knowledge, there has been no information on the clinical significance of EF-1 delta mRNA expression in malignant tumours, such as oesophageal carcinoma. Oesophageal carcinoma has proven to be one of the most difficult malignancies to cure, and the prognosis for these patients has been extremely poor (Roth *et al*, 1997). In contrast, long-term survival was recognised in some patients who underwent a curative operation, and it is therefore very important for surgeons and gastroenterologists to identify effective markers for postoperative prognosis. Consequently, we quantitated the expression of EF-1 delta in malignant and benign oesophageal tissues and investigated whether these levels were associated with clinicopathologic parameters and the prognosis in cases of oesophageal carcinoma.

## MATERIALS AND METHODS

### Patients and sample collection

Elongation factor-1 delta mRNA expression levels were investigated in a series of 52 oesophageal carcinoma specimens from patients who underwent curative surgery at the Medical Institute of Bioregulation Hospital, Kyushu University and Saitama Cancer Center. All 52 patients were clearly identified based on clinicopathologic findings. No patients received chemotherapy or radiotherapy prior to the operation. The patients included 47 males and five females. Patient ages ranged from 40 to 82 years with a median age of 63 years. In all, 10 tumours were well differentiated, 27 were moderately differentiated, and eight were poorly differentiated squamous cell carcinomas. Other types of carcinomas included basal cell carcinoma (*n*=3), adenocarcinoma (*n*=2) and small-cell carcinoma (*n*=2). The depth of tumour invasion was as follows: five involving the submucosa, nine involving the muscularis propria and 38 in the adventitia or deeper. Patients with lymph node metastases were classified into two groups: the nonmetastatic group (*n*=12) and the metastatic group (*n*=40). Carcinoma specimens were obtained from the tumour edge, avoiding the necrotic centre, and corresponding distant normal mucosa specimens were also obtained at least 5 cm away from the tumour edge by sharply dissecting the mucosa off the muscularis propria. All specimens were immediately frozen in liquid nitrogen and kept at −80°C until the extraction of RNA. Written informed consent was obtained from all patients.

### Total RNA extraction

Frozen tissue specimens or cultured cells in a state of subconfluency (human oesophageal cancer cell line KYSE30) were homogenised in guanidinium thiocyanate, and total RNA was obtained by ultracentrifugation through a cesium chloride cushion as described previously (Mori *et al*, 1993; Yoshinaga *et al*, 2003). KYSE30 was kindly provided by Dr Shimada (First Department of Surgery, Faculty of Medicine, Kyoto University, Kyoto, Japan).

### Real-time quantitative reverse transcription–polymerase chain reaction (RT–PCR)

The cDNA was synthesised from 8.0 *μ*g of total RNA as described previously (Inoue *et al*, 1995; Yamashita *et al*, 2001). Two gene-specific oligonucleotide primers were designed:

sense EF-1 delta, 5′-CCCGCGTCCGCCGATTCCTC-3′;
antisense EF-1 delta, 5′ CGCTGGCGCCGTTCTCCTG-3′;
sense glyceraldehyde 3-phosphate dehydrogenase (GAPDH), 5′-TTGGTATCGTGGAAGGACTCA-3′;
antisense GAPDH, 5′-TGTCATCATATTTGGCAGGTT-3′.

These primers spanned more than two exons to avoid amplification of any contaminating DNA.

Real-time monitoring of PCR reactions was performed using the LightCycler™ system (Roche Applied Science, Indianapolis, IN, USA), and SYBR green I dye (Roche Diagnostics), which binds preferentially to double-stranded DNA, according to the manufacturer's instructions. Fluorescent signals are proportional to the concentration of the product and are measured at the end of each cycle, rather than after a fixed number of cycles (Buckhaults *et al*, 2001). The higher the starting quantity of template, the earlier the attainment of the threshold cycle, which is defined as the fractional cycle number at which fluorescence passes a fixed threshold above baseline (Bieche *et al*, 1998, 1999).

For cDNAs of the 52 paired oesophageal samples (cancerous tissues and corresponding normal counterparts), the PCR reaction was carried out on the Light Cycler system. For each run, a master mixture, containing 1 *μ*l of cDNA, 2 *μ*l of LC DNA Master SYBR Green I mix, 50 ng of primers and 2.4 *μ*l of 25 mM MgCl_2_, was prepared on ice. The final volume was adjusted to 20 *μ*l with water. After the reaction mixture was loaded into the glass capillary tube, the cycling conditions were carried out as follows: initial denaturation at 95°C for 10 min, followed by 40 cycles of denaturation at 95°C for 10 s, annealing at 62°C (60°C for GAPDH) for 10 s and extension at 72°C for 10 s. The temperature transition rate was set at 20°C s^−1^.

For distinguishing specific from nonspecific products and primer dimers, a melting curve was obtained after amplification by holding the temperature at 68°C for 30 s, and then gradually increasing the temperature to 95°C at a rate of 0.2°C s^−1^. No amplification of nonspecific products was observed. To verify the melting curve results, each representative sample of the PCR products was run on 1.5% agarose gels, and a single PCR product of the size predicted from the cDNA was confirmed.

We determined the levels of EF-1 delta and GAPDH mRNA expression by comparisons with cDNA from the human oesophageal cancer cell line KYSE30. After proportional baseline adjustment, the fit point method was employed to determine the cycle in which the log-linear signal was distinguished from the baseline, and that cycle number (threshold cycle) was used as a crossing-point value. The standard curve was produced by measuring the crossing point of each standard value (four-fold serially diluted cDNAs of KYSE30), and plotting them against the logarithmic value of concentrations. The standard curve samples were included in each run. The concentrations of each sample were then calculated by plotting their crossing points against the standard curve. All calculated concentrations are relative to the concentration of the cDNA of KYSE30, and the amount of target molecule was then divided by the amount of the endogenous reference (GAPDH), to obtain normalised EF-1 delta expression (Bieche *et al*, 1999). Each assay was performed at least twice to verify the results, and the mean mRNA expression was used for analysis.

### Statistical analysis

The median follow-up time for the 12 living patients was 8.0 years (range: 5.1–11.4 years). Overall and cause-specific survival rates were calculated actuarially according to the Kaplan–Meier method (Kaplan and Meier, 1958), and were measured from the day of surgery. Differences between groups were estimated using the *χ*^2^ test, the Student's *t* test, and the log-rank test (Mantel, 1966). A probability level of 0.05 was chosen for statistical significance. Statistical analysis was performed with the SPSS software package (version 6.1; SPSS, Inc., Chicago, IL, USA).

## RESULTS

**Figure 1:**
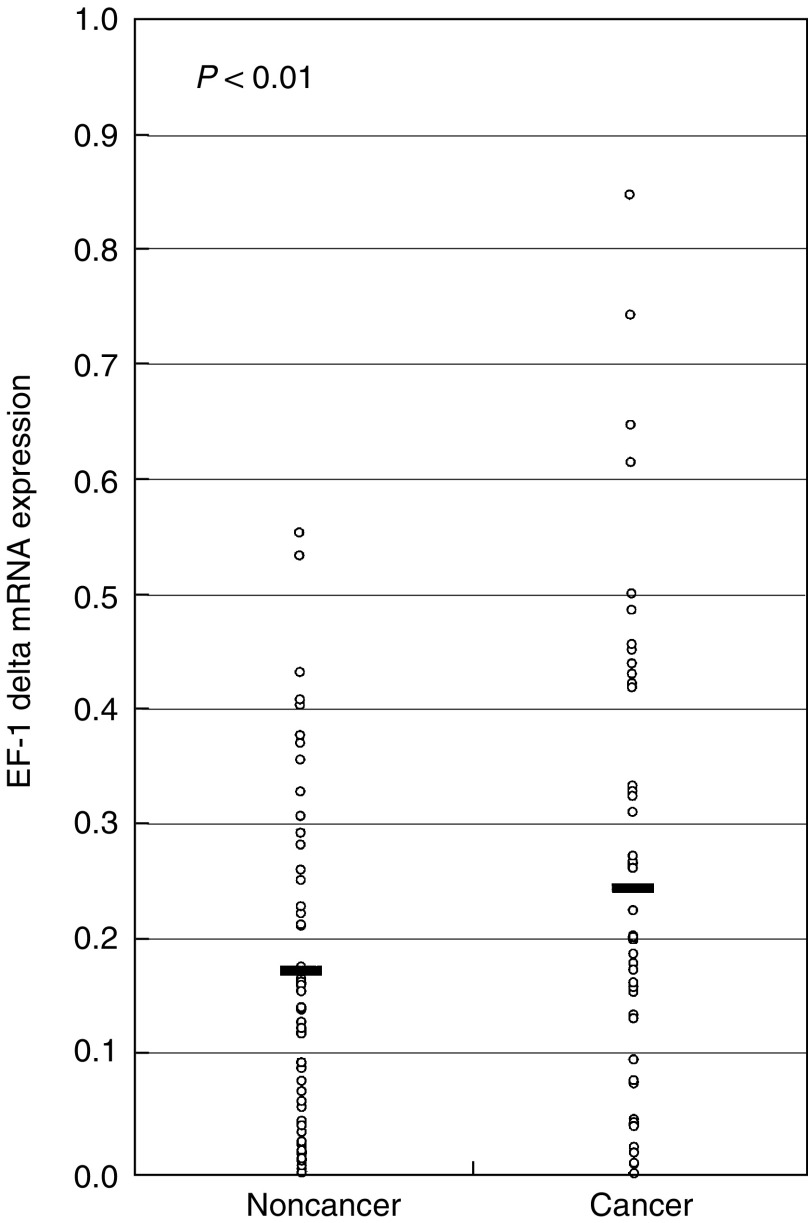
Elongation factor-1 delta expression in cancerous and noncancerous oesophageal tissues. The horizontal lines indicate the means. Cancer tissues showed significantly higher EF-1 delta mRNA expression levels as compared to noncancerous tissues (*P*<0.01). The *P*-value was calculated by the Student's *t*-test.

With regard to EF-1 delta mRNA expression in clinical samples, 38 of 52 patients (73%) showed a higher expression level of EF-1 delta mRNA in cancerous tissues in comparison with noncancerous tissues by real-time quantitative RT–PCR. The mean expression level of EF-1 delta mRNA in tumour tissues, 0.250±0.200 (mean±standard deviation (s.d.)), was significantly higher than 0.178±0.151 in the corresponding normal tissues (*P*<0.01, Figure 1

). The median expression levels of EF-1 delta mRNA in tumour tissues and normal tissues were 0.198 and 0.151, respectively. In the current study, the patients with values less than the median expression level (0.198) in tumour tissues were considered to be in the low expression group (*n*=26), whereas those with values ⩾0.198 were considered to be in the high expression group (*n*=26). Table 1

**Table 1:** Clinicopathologic data and elongation factor-1 delta mRNa expression in the tumour specimens of 52 patients with oesophageal carcinoma

	**High expression^b^**	**Low expression^b^**	
**Variable**	***n*=26**	***n*=26**	***P-*value**
Age	63.9±9.4	59.3±8.6	0.069
Gender (male)			
Male	25	22	0.158
Female	1	4	
Histological grade^a^			
Well	4	6	0.901
Moderately	14	13	
Poorly	4	4	
Others	4	3	
Depth of invasion			
Submucosa	1	4	0.525
Muscularis propria	4	5	
Adventitia	21	17	
Lymph node metastasis			
Absent	3	9	**0.032**^d^
Present	24	16	
Lymphatic permeation			
Absent	0	3	0.089
Present	26	23	
Venous permeation			
Absent	3	7	0.159
Present	23	19	
Clinical stage^c^			
I, II	6	14	**0.023**^d^
II, IV	20	12	

a This analysis included 45 squamous cell carcinomas and others including basal cell carcinoma (*n*=3), adenocarcinoma (*n*=2) and small-cell carcinoma (*n*=2).

b Low and high groups were determined by a median value of elongation factor-1 delta mRNA (0.198).

c According to the International Union Against Cancer 1997 TNM classification system.

d Statistically significant.

shows the clinicopathologic data and EF-1 delta mRNA expression in the tumour specimens from 52 patients with oesophageal carcinoma. The incidence of lymph node metastasis in the high expression group (24 of 26, 92%) was significantly higher (*P*=0.032) than that in the low expression group (16 of 26, 62%). Moreover, the incidence of advanced stages (according to the International Union Against Cancer 1997 TNM classification system) in the high expression group (20 of 26, 77%) was significantly higher (*P*=0.023) than that in the low expression group (12 of 26, 46%).

**Figure 2:**
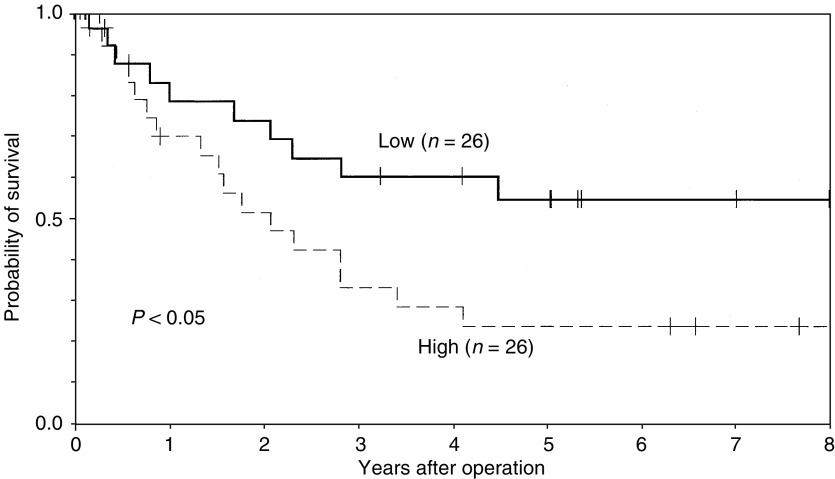
Actuarial cause-specific survival in patients with oesophageal carcinoma according to the level of EF-1 mRNA expression.

In all, 40 of 52 patients (77%) died during the period of this analysis. A total of 27 patients died of oesophageal carcinoma, and the remaining 13 patients died without any sign of clinical recurrence (such as pneumonia and cerebrovascular diseases). The 5-year actuarial overall survival rates in patients with higher EF-1 mRNA levels and those with lower EF-1 mRNA levels were 19 and 42%, respectively. The difference between these two groups was not statistically significant (*P*=0.065). On the other hand, the 5-year actuarial cause-specific survival rates were 23% for patients with high EF-1 delta mRNA levels and 54% for those with low EF-1 delta mRNA levels (Figure 2

), and this was significantly different (*P*=0.046).

## DISCUSSION

The results of the current study indicated that EF-1 delta mRNA overexpression in cancerous compared to noncancerous tissue is commonly observed in patients with oesophageal carcinoma. Moreover, the higher mRNA expression of EF-1 delta was significantly associated with lymph node metastases and advanced disease stages. Similar findings have also been reported in gastric and colorectal cancer patients with a higher expression of EF-1 gamma (Chi *et al*, 1992; Mimori *et al*, 1995). We have also demonstrated that oesophageal cancer patients with EF-1 gamma overexpression disclosed severe lymph node metastases and many of these patients were found to be in the most advanced stages (Mimori *et al*, 1996).

The current study also indicated that a higher expression of EF-1 delta mRNA was correlated with poorer prognosis for patients with oesophageal carcinoma. With regard to EF-1 delta expression in cancers, Kolettas *et al* (1998) indicated that EF-1 delta was more highly expressed in cDNA from the original cancer cell population than in normal cells by Southern-plaque hybridisation. Shuda *et al* (2000) found that the mRNA expression of EF-1 delta was upregulated along with the histological grading (from well, moderately, to poorly differentiated) in hepatocellular carcinoma. The higher expression of EF-1 delta in the tumours suggested that malignant transformation *in vivo* requires an increase in translation factor mRNA and protein synthesis for entry into and transition through the cell cycle (Kolettas *et al*, 1998). Similarly, others have indicated that EF-1 alpha and EF-1 gamma were highly expressed in certain tumours including colon, lung, gastric, pancreatic and breast tumours, relative to normal adjacent tissue (Chi *et al*, 1992; Grant *et al*, 1992; Lew *et al*, 1992; Ender *et al*, 1993; Mathur *et al*, 1998; Su *et al*, 1998). These results suggest that alteration in the expression of EF-1, including EF-1 delta, contribute to cell transformation and cancer development.

More recent reports have indicated that EF-1 delta can function as a proto-oncogene when overexpressed (Joseph *et al*, 2002; Lei *et al*, 2002). Joseph *et al* (2002) indicated that overexpression of EF-1 delta protein by transfection was oncogenic in NIH3T3 cells, as evidenced by the appearance of transformed foci exhibiting anchorage-independent growth and the potential to grow as tumours in nude mice. They also demonstrated that cell transformation and tumorigenesis induced by cadmium are due, at least in part, to the overexpression of EF-1 delta. Lei *et al* (2002) found that blocking EF-1 delta with antisense mRNA resulted in a significant reversal of its oncogenic potential. These results suggest that EF-1 delta may function as a potential proto-oncogene when overexpressed. Therefore, EF-1 delta may be used as a potential therapeutic target or as a risk factor for carcinogenesis in tumours that exhibit its overexpression.

Elongation factor-1 delta may have other actions, such as effects on chemotherapy and ionising radiation (Jung *et al*, 1994; Sinha *et al*, 2000). Sinha *et al* (2000) indicated that human EF-1 delta was overexpressed in melanoma cell lines exhibiting chemoresistance towards antineoplastic drugs, such as vindesine, cisplatin, fotemstine and etoposide. They suggested that the development of chemoresistance towards anti-neoplastic drugs in melanoma cell lines could be the results of a changed functionality of the general translation machinery. With regard to ionising radiation, Jung *et al* (1994) indicated that EF-1 delta was a radiation-inducible gene and might participate in the G2-M cell cycle checkpoint. Further functional studies (e.g., proliferation assays, transfection experiments) should be undertaken to clarify the role of EF-1 delta mRNA in chemoresistance and radiation-inducible gene expression.

In the current study, 38 of 52 oesophageal carcinoma patients (73%) showed overexpression of EF-1, while the remaining 14 patients (27%) did not exhibit an overexpression of EF-1. These results suggest that the etiological factors or the molecular mechanisms responsible for oesophageal carcinogenesis may be different for those tumour samples overexpressing EF-1 delta compared to those not overexpressing EF-1 delta. We have previously indicated that loss of fragile histidine triad (Fhit) expression may be an early event in the development of human oesophageal carcinoma and patients who were both heavy users of tobacco and alcohol showed a significantly higher frequency of loss of Fhit expression than those who were light users (Mori *et al*, 2000). Since EF-1 delta has been proven to be a cadmium-responsive proto-oncogene (Joseph *et al*, 2002), the potential etiological factors, such as exposure to certain chemical carcinogens and lifestyle (smoking and alcoholism), may also affect the expression of EF-1 delta in patients with oesophageal carcinoma, as was observed with Fhit expression. However, the precise mechanisms of EF-1 delta overexpression in oesophageal carcinoma are uncertain. The amplification of the genomic locus of EF-1 delta, located on chromosome 8q24.3, or the promotion of gene transcription may explain EF-1 delta overexpression. Further studies are required to elucidate the possible mechanisms of EF-1 delta overexpression in oesophageal carcinoma.

In conclusion, our results indicated that EF-1 mRNA was overexpressed in oesophageal carcinoma tissues, and higher expression levels were correlated with lymph node metastasis, advanced disease stages and poor prognosis. These findings suggest a possible role for EF-1 delta expression as an additional diagnostic and prognostic biomarker for oesophageal carcinoma. Furthermore, understanding the biological function of EF-1 delta expression in oesophageal tissue may help to delineate its role in oesophageal physiology and the pathology of oesophageal carcinoma.
